# Supplementary material for: Safely managed sanitation practice and childhood stunting among under five years old children in Myanmar
Source: PLoS One. 2023 Nov 20;18(11):e0290600. doi: 10.1371/journal.pone.0290600 (PMC10659194; doi:10.1371/journal.pone.0290600)
Supplement: S1 File — (DOCX) [file pone.0290600.s001.docx]

Participant ID

Date -----------/----------/--------------- (dcd)

**QUESTIONNAIRES**

**Safely managed sanitation practices and childhood stunting among under five years old children in Myanmar**

Please circle the answer or fill in the blanks for explanation the truth.

**Part 1. Social Demographic Factors**

| **Information** | | For researcher |
| --- | --- | --- |
| 1. | **Place of residence: Township**  1. Mohnyin 2. Myitkyina 3. Mudon 4. Ye’  5. Ramee 6. MraukU 7. Pindaya 8. Lashio  9. Kyaiklat 10. Kyankhin 11. Seikphyu 12. Magway  13. Myitthar 14 Kyaukpadaung 15. Dawei 16. Myeik | a1 ------ |
| 2 | **Place of residence: Urban/Rural**  1. Urban 2. Rural | a2 ------ |
| 3 | **Kitchen location**  1. Outside home 2. Attached home 3. Inside home | a3 ------ |
| 4 | **Having poultry animals: Inside house**  1. No 2. Yes | a4------ |
| 5 | **If yes, animal feces can see inside house? (Observe)**  1. No 2. Yes | a5------ |
| 6 | **House floor type**  1. Concrete 2. Wood 3. Earth | a6 ------- |
| 7 | **Family size number ----------------------** | a7 ------- |

**Part 2. Child Factors**

| **Information** | | For researcher |
| --- | --- | --- |
| 1. | **Date of Birth ----/-------/-------- (-------- months)** | b1 ------ |
| 2 | **Gender** 1. Female 2. Male | b2 ------ |
| 3 | **Parity ------------------** | b3 ------ |
| 4 | **Parity number -----------------** | b4 ------ |
| 5 | **Gestational age --------------- months -------------days** | b5 ------- |
| 6 | **Birth weight -------------kg** | b6 ------- |
| 7 | **Birth interval ------------- years ------------ months** | b7 ------ |
| 8 | **Vitamin A supplement** 1. No 2. Yes | b8 ------ |
| 9 | **Deworming** 1. No 2. Yes 3. No, child is under 1 year old | b9 ------ |
| 10 | **Antenatal Care visit times during pregnancy ----------------- times** | b10 ------ |
| 11 | **a. Breast Feeding**  1. No 2. Yes  **b. If no, what type of feeding?**  1. Animal milk 2. Milk Powder 3. Others -------------------------------  **c. If breast fed, did you do exclusive breast feeding until 6 month of child’s age?**  1. No 2. Yes  **d. When did you do the complementary food feeding to your child?**  ------------- month | b11 ------ |

**Part 3. Parent factors**

| **Information** | | | | | | | | | | For researcher |
| --- | --- | --- | --- | --- | --- | --- | --- | --- | --- | --- |
| 1. | **Mother’s age**  **Date of Birth Mother ----/-----/------- Or (--------years)** | | | | | | | | | c1 ------ |
| 2 | **Educational Attainment - Mother**  1. University graduate 2. High School 3. Middle school  4. Primary School 5. Can read and write 6. Illiterate | | | | | | | | | c2 ------ |
| 3 | **Occupation - Mother**  1. Government staff 2. Private employee 3. Dependent  4. Manual labor 5. Own business 6. Others specify------------------- | | | | | | | | | c3 ------ |
| 4 | **Occupation - Father**  1. Government staff 2. Private employee 3. Dependent  4. Manual labor 5. Own business 6. Others specify------------------- | | | | | | | | | c4 ------ |
| 5 | **Monthly Family income (MMK) ----------- Kyats** | | | | | | | | | c5 -------- |
| 6 | **Monthly Family income (MMK) converted to ----------- USD** | | | | | | | | | c6-------- |
| 7 | **Care giver**  1. Parent 2. Mother 3. Father 4. Grand Mother/Grand Father 5. Relatives | | | | | | | | | c7 -------- |
| 8 | **Smoking- Mother**  1. No 2. Yes | | | | | | | | | c8 ------ |
| 9 | **Smoking- Father**  1. No 2. Yes | | | | | | | | | c9 ------ |
| 10 | **Smoking- Any other household member**  1. No 2. Yes | | | | | | | | | c10 ------ |
| 11 | **Decision on food is made by**  1. Mother 2. Father 3. Grand Father/Mother 4. Others ----------- | | | | | | | | | c11 -------- |
| 12 | **Mother’s knowledge on childhood malnutrition** | | | | | | | | |  |
|  | **Information** | | | **Yes** | | **No** | | **Don’t Know** | |  |
| a | Imbalanced diet is the cause of malnutrition | | |  | |  | |  | |  |
| b | Less weight/height for age is a clinical feature of malnutrition | | |  | |  | |  | |  |
| c | Milk, meat, grains and vegetables are essential for the growth of children | | |  | |  | |  | |  |
| d | Breastfeeding should be initiated within half an hour of delivery. | | |  | |  | |  | |  |
| e | Supplementary feeding should be initiated at 6 months of age | | |  | |  | |  | |  |
| f | Delayed physical growth and impaired cognitive development are the complication of malnutrition | | |  | |  | |  | |  |
| g | Regular deworming will prevent malnutrition in children | | |  | |  | |  | |  |
| h | Immunization of children is the best way to protect the child against infectious disease and malnutrition | | |  | |  | |  | |  |
| i | Every 3 months, height and weight must be checked to under 5 years old children | | |  | |  | |  | |  |
| j | Adequate breast feeding and nutritious food will prevent malnutrition in children | | |  | |  | |  | |  |
|  | **Total score** | | | **---------------------** | | | | | |  |
|  | **Knowledge percentage** | | | **---------------------** | | | | | | c12 ------ |
| 13 | **Mother’s attitude on childhood malnutrition** | | | | | | | | |  |
|  | **Information** | **Strongly disagree** | **Disagree** | | **Neutral** | | **Agree** | | **Strongly Agree** |  |
| a | Supplementary food including water should be provided only after 6 months age |  |  | |  | |  | |  |  |
| b | Giving different types of food is beneficial to child’s growth |  |  | |  | |  | |  |  |
| c | Feeding child several times each day is beneficial for child’s development |  |  | |  | |  | |  |  |
| d | Exclusive breast feeding until 6 month age of children is critical for child growth |  |  | |  | |  | |  |  |
| e | For the sake of child’s physical development, mother should avoid some meats or vegetables or fruits during pregnant and breast feeding period |  |  | |  | |  | |  |  |
| f | Practicing improved toilet and its cleanliness is not beneficial for childhood nutrition |  |  | |  | |  | |  |  |
| g | Washing hand with soap and water is not beneficial for childhood nutrition |  |  | |  | |  | |  |  |
| h | Child growth is normal phenomenon, it is not depending on the food intakes and other factors |  |  | |  | |  | |  |  |
| i | Malnutrition is unpreventable disease, it is a genetic problem |  |  | |  | |  | |  |  |
| j | Children’s weight and height is totally depending on parent’s weight and height, it doesn’t relate with child’s food pattern |  |  | |  | |  | |  |  |
|  | **Total score** | **---------------------** | | | | | | | |  |
|  | **Attitude percentage** | **---------------------** | | | | | | | | c13 ------ |

**Part 4. WASH practices**

| **Information** | | For researcher |
| --- | --- | --- |
| 1. | **Water: What is the main source of drinking water for the members of your household?**   1. Piped water   (11. □ Piped into dwelling 12. □ Piped into compound, yard or plot  13. □ Piped to neighbor 14. □ Public tap / standpipe 15. □ Borehole or tube-well)   1. Dug well (21. □ Protected well 22. □ Unprotected well) 2. Water from spring (31. □ Protected spring 32. □ Unprotected spring   33. □ Rainwater collection)   1. Delivered water (41. □ Tanker-truck 42. □ Cart with small tank / drum) 2. Water Kiosk 3. Packaged water (61. □Bottled water 62. □ Sachet water) 4. Surface water (71. □ river 72. □ stream 73. □ dam 74. □ lake 75. □ pond 76. □ canal 77. □ irrigation channel) 5. Other (specify) -------------------- | d1 -------- |
| 2 | **Where is that water collected from?**  1. In own dwelling 2. In own yard / plot 3. Elsewhere | d2 -------- |
| 3 | **How long does it take to go there, get water, and come back?**  **00. Members do not collect Number of minutes …………** | d3 -------- |
| 4 | **In the last year, has there been any time when your household did not have sufficient quantities of drinking water when needed?**  1. Yes, at least once 2. No, always sufficient 3. Don’t know | d4 -------- |
| 5 | **Drinking water services level (To complete by researcher)**  1. At least basic water supply 2. Limited 3. Unimproved 4. Surface water | d5 -------- |
| 6 | **Sanitation: What kind of toilet facility do members of your household usually use?**   1. Flush / pour flush (11. □ Flush to piped sewer system 12. □Flush to septic tank 13. □ Flush to pit latrine 14. □ Flush to open drain 15. □Flush to don’t know where) 2. Dry pit latrines (21. □ Pit latrine with slab 22. □ Pit latrine without slab / Open pit ) 3. Composting toilets (31. □ Twin pit with slab 32. □ Twin pit without slab   33. □ Other composting toilet)   1. □ Bucket 2. □ Container based sanitation 3. □ Hanging toilet / hanging latrine 4. □ No facility / Bush / Field 5. □ Other (specify) ---------------- | d6 ------ |
| 7 | **Do you share this facility with others who are not members of your household?**  1. No 2. Yes | d7 ------ |
| 8 | **Where is this toilet facility located?**  **1.** In own dwelling  **2.** In own yard / plot  **3.** Elsewhere | d8 ------ |
| 9 | **Has your (pit latrine or septic tank) ever been emptied?**  1. Yes emptied 2. Never emptied 3. Don’t know | d9 ------ |
| 10 | **The last time if it was emptied, how did you managed and where were the contents emptied to?**   1. Removed by service provider (11. □ to a treatment plant 12. □ buried in a covered pit 13. □ don’t know where to) 2. Emptied by household (21. □ buried in a covered pit 22. □ to uncovered pit, open ground, water body or elsewhere) 3. □ Other (specify) ------------------ 4. □ Don’t know 5. □ Don’t have toilet | d10 ------ |
| 11 | **What was done to dispose of the child stools?**  1. Child used toilet/latrine  2. Put/rinsed into toilet or latrine  3. Put/rinsed into drain or ditch  4. Thrown into garbage  5. Buried  6. Left in the open  7. Used as manure (mixed with animal’s excreta)  8. Other (specify)----------------  9. Don’t know | d11 ------ |
| 12 | **Is fecal containment safe? Check the below box after observation:**  Away from water source at least 50 feet  Toilet pit was built up with concrete ring or brick with cement  No damage/leakage in toilet pit, pipe and floor | d12 ------ |
| 13 | **Sanitation services level**  1. Safely managed 2. Basic 3. Limited  4. Unimproved 5. Open defecation | d13 ------ |
| 14 | **Hygiene: Can you show where do you and other members of your household most often wash your hands?**  1. Fixed facility reported (sink/tap) (□ In dwelling □In yard/plot)  2. Mobile object reported (bucket/jug/kettle)  3. No handwashing place in dwelling/yard/plot  4. No permission to see  5. Other (specify) ---------------------- | d14 ------ |
| 15 | **Water available at handwashing facility** 1. No 2. Yes | d15 ------ |
| 16 | **Soap available at handwashing facility** 1. No 2. Yes | d16 ------ |
| 17 | **Hygiene services level**  1. Basic 2. Limited 3. No handwashing facility | d17 ------- |
| 18 | **How does your household usually dispose of garbage?**  1. Collected by formal service provider  2. Collected by informal service provider  3. Disposed of in designated waste disposal area  4. Disposed of within household yard or plot  5. Buried or burned  6. Disposed of elsewhere  7. Don’t know | d18 ------- |
| 19 | **How do you dispose of household water used for cooking, laundry and bathing?**  1. Sink/drain connected to sewer  2. Sink/drain connected to septic tank  3. Sink/drain connected to pit  4. Sink/drain connected to soak pit  5. Sink/drain connected to open drain or open ground  6. Disposed directly to water body  7. N/A (cooking, laundry and bathing is done away from the household)  8. Don’t know | d19 ------- |
| 20 | **Mother hand washing with soap and water with systematic method (Observation)**  1. Yes No, ----------- steps included | d20 -------- |
| 21 | **Father hand washing with soap and water with systematic method (Observation)**  1. Yes No, ----------- steps included | d21 -------- |
| 22 | **Caregiver’s hand washing with soap and water with systematic method (Observation)**  1. Yes No, ----------- steps included | d22 -------- |
| 23 | **Child’s hand washing with soap and water with systematic method (Observation)**  1. Yes No, ----------- steps included | d23 -------- |

**Part 5. Food safety and diet pattern**

| **Information** | | For researcher |
| --- | --- | --- |
| 1 | **Food safety facts**  **Clean**  Washing hand with soap before cooking  1. Never 2. Sometime 3. Ever  Washing foods with water before cooking at least 20 seconds  1. Never 2. Sometime 3. Ever  **b. Contain**  Contain meats and vegetables separately while carrying from market to home  1. Never 2. Sometime 3. Ever  Contain meats and vegetables separately at kitchen  1. Never 2. Sometime 3. Ever  **c. Cook**  Cook the meats and vegetable with fully boiled  1. Never 2. Sometime 3. Ever  Cook the meats and vegetable with fully fried  1. Never 2. Sometime 3. Ever  **d. Chill**  Store the foods in refrigerator  1. Never 2. Sometime 3. Ever  Foods are cooked daily basis  1. Never 2. Sometime 3. Ever | e1a --------  e1b --------  e1c --------  e1d --------  e1e --------  e1f --------  e1g --------  e1h -------- |
| 2 | **Food safety score ---------------------------** | e2 -------- |
| 3 | **Main Food**  1. Breastmilk, 2. Rice 3. Others ------------- | e3 -------- |
| 4 | **Accessory Foods (Diet diversity)**  1. Dairy products (Milk, Cheese, Yogurt)  2. Meats (Chicken, Pork, Beef, Mutton, Fish)  3. Egg  4. Beans  5. Grains, Roots and Tubers  6. Fruits  7. Vegetables  8. Legume and Nuts | e4 -------- |
| 5 | **What kinds of foods are avoided since birth?**  1. No 2. Yes | e5 -------- |

**Part 6. Health Literacy**

**Sanitation Promotion Health literacy**

| No | Information | Very difficult  1 | Difficult  2 | Easy  3 | Very easy  4 | Don’t know |
| --- | --- | --- | --- | --- | --- | --- |
| 1 | Find information the importance of using sanitary latrine |  |  |  |  |  |
| 2 | Find information how to build, operate and maintain the sanitary latrine |  |  |  |  |  |
| 3 | Understand the sanitation promotion information that was given by health personals and other communication channels such as radio, TV, Facebook, etc., |  |  |  |  |  |
| 4 | Understand sanitation promotion messages these are described on printed media such as pamphlet, billboard, poster, etc., |  |  |  |  |  |
| 5 | Judge the sanitation promotion information that was given by health personals and other communication channels such as radio, TV, Facebook, etc., |  |  |  |  |  |
| 6 | Judge sanitation promotion messages these are described on printed media such as pamphlet, billboard, poster, etc., |  |  |  |  |  |
| 7 | Use the sanitary latrines according the hygiene promotion messages |  |  |  |  |  |
| 8 | Renovate and maintain the existing latrine according to sanitation promotion messages |  |  |  |  |  |
|  | | f1------------- | | | | |

**Hygiene Promotion Health literacy**

| No | Information | Very difficult  1 | Difficult  2 | Easy  3 | Very easy  4 | Don’t know |
| --- | --- | --- | --- | --- | --- | --- |
| 1 | Find information the importance of hand washing with water and soaps properly |  |  |  |  |  |
| 2 | Find information how to build, operate and maintain the hand washing facilities |  |  |  |  |  |
| 3 | Understand the hygiene promotion information that was given by health personals and other communication channels such as radio, TV, Facebook, etc., |  |  |  |  |  |
| 4 | Understand hygiene promotion messages these are described on printed media such as pamphlet, billboard, poster, etc., |  |  |  |  |  |
| 5 | Judge the hygiene promotion information that was given by health personals and other communication channels such as radio, TV, Facebook, etc., |  |  |  |  |  |
| 6 | Judge hygiene promotion messages these are described on printed media such as pamphlet, billboard, poster, etc., |  |  |  |  |  |
| 7 | Wash hands in 4 critical times with 7 steps according the hygiene promotion messages |  |  |  |  |  |
| 8 | Renovate and maintain the existing hand washing space/facilities according to hygiene promotion messages |  |  |  |  |  |
|  | | f2------------ | | | | |

**Nutrition promotion health literacy**

| No | Information | Very difficult  1 | Difficult  2 | Easy  3 | Very easy  4 | Don’t know |
| --- | --- | --- | --- | --- | --- | --- |
| 1 | Find information about complementary food feeding for children |  |  |  |  |  |
| 2 | Find information about the nutritious food for children |  |  |  |  |  |
| 3 | Understand the nutritional information that was given by health personals and other communication channels such as radio, TV, Facebook, etc., |  |  |  |  |  |
| 4 | Understand nutrition promotion messages these are described on printed media such as pamphlet, billboard, poster, etc., |  |  |  |  |  |
| 5 | Judge the nutrition promotion information that was given by health personals and other communication channels such as radio, TV, Facebook, etc., |  |  |  |  |  |
| 6 | Judge nutrition promotion messages these are described on printed media such as pamphlet, billboard, poster, etc., |  |  |  |  |  |
| 7 | Feed complementary food after 6 months age of children |  |  |  |  |  |
| 8 | Prepare foods to be nutritious according to nutritional promotion messages |  |  |  |  |  |
|  | | f3--------------------------- | | | | |

**Part 7. Disease factors**

| **Information** | | For researcher |
| --- | --- | --- |
| 1. | **Had diarrhea in the past 2 weeks** (three or more loose or liquid bowel movements over a 24 hour)  1. No 2. Yes | g1 ------ |
| 2 | **Had dysentery in the past 2 weeks** (diarrhea with blood or mucus with abdominal pain over a 24 hour)  1. No 2. Yes | g2 ------ |
| 3 | **Had worm infection in the past 2 weeks** (abdominal pain with itchy anus and unexplained weight loss over a 24 hour)  1. No 2. Yes | g3 ------ |
| 4 | **Frequency of sickness (get health care services for any sickness)**  1. No 2. Yes | g4 ------ |

**Part 8. Anthropometric Measurement**

| No | Information | For researcher |
| --- | --- | --- |
| 1 | **Mother’s height in cm** -------------cm | h1 ------ |
| 2 | **Father’s height in cm** -------------cm | h2 ------ |
| 3 | **Child’s height in cm** -------------cm | h3 ------ |
