# Supplementary material for: Safely managed sanitation practice and childhood stunting among under five years old children in Myanmar
Source: PLoS One. 2023 Nov 20;18(11):e0290600. doi: 10.1371/journal.pone.0290600 (PMC10659194; doi:10.1371/journal.pone.0290600)
Supplement: Attachment — Submitted filename: Response to reviewers.docx [file pone.0290600.s002.docx]

Dear Ranjit,

I would like to express my gratitude for the time and effort you and the reviewers have invested in the evaluation of our manuscript, " Safely managed sanitation practice and childhood stunting among under five years old children in Myanmar," manuscript ID: PONE-D-23-25415. We are sincerely appreciative of the insightful feedback provided, which has significantly improved the quality of our work. I would like to respond to the reviewers' comments and outline the revisions we have made.

**Editor comments:**

1. Please ensure that your manuscript meets PLOS ONE's style requirements, including those for file naming. The PLOS ONE style templates can be found at

<https://journals.plos.org/plosone/s/file?id=wjVg/PLOSOne_formatting_sample_main_body.pdf> and

<https://journals.plos.org/plosone/s/file?id=ba62/PLOSOne_formatting_sample_title_authors_affiliations.pdf>

**Ans:** Author contribution information has been added in title page to be align with PLOSONE structure guideline. And line numbers are added to manuscript file. Manuscript body meets with PLOS ONE's style requirements.

2. We note that you have indicated that data from this study are available upon request. PLOS only allows data to be available upon request if there are legal or ethical restrictions on sharing data publicly. For more information on unacceptable data access restrictions, please see <http://journals.plos.org/plosone/s/data-availability#loc-unacceptable-data-access-restrictions> .

Ans: The data has been deposited in DOI provided data repository. The link is below: <https://datadryad.org/stash/share/2PvdlnZW-nGe5WYZ_Iq1vfNeZC6DL1nShFSibf9o1B8>

In your revised cover letter, please address the following prompts:

a) If there are ethical or legal restrictions on sharing a de-identified data set, please explain them in detail (e.g., data contain potentially sensitive information, data are owned by a third-party organization, etc.) and who has imposed them (e.g., an ethics committee). Please also provide contact information for a data access committee, ethics committee, or other institutional body to which data requests may be sent.

Ans: there is no any ethical or legal restriction on sharing this data.

b) If there are no restrictions, please upload the minimal anonymized data set necessary to replicate your study findings as either Supporting Information files or to a stable, public repository and provide us with the relevant URLs, DOIs, or accession numbers. For a list of acceptable repositories, please see http://journals.plos.org/plosone/s/data-availability#loc-recommended-repositories.

Ans: The data has been deposited in DOI provided data repository. The link is below: <https://datadryad.org/stash/share/2PvdlnZW-nGe5WYZ_Iq1vfNeZC6DL1nShFSibf9o1B8>

We will update your Data Availability statement on your behalf to reflect the information you provide.

Ans: Thank you so much for your support and kind understanding on that.

3. Your ethics statement should only appear in the Methods section of your manuscript. If your ethics statement is written in any section besides the Methods, please move it to the Methods section and delete it from any other section. Please ensure that your ethics statement is included in your manuscript, as the ethics statement entered into the online submission form will not be published alongside your manuscript.

Ans: Thank your for your suggestion. Ethical consideration and consent to participate statement has been moved to method section. And it has been removed from additional information part.

4. Please include captions for your Supporting Information files at the end of your manuscript, and update any in-text citations to match accordingly. Please see our Supporting Information guidelines for more information: <http://journals.plos.org/plosone/s/supporting-information> .

Ans: Caption has been revised to supporting documents to match with supporting information guideline.

**Reviewer 1 Comments:**

I suggest changing the title to "Prevalence and factors associated with stunting among children under five years in Myanmar”. Safely managed sanitation practice is not the only variable found associated with stunting."

Ans: Yes, well noted that and thanks. Actually, this study focus to answer “Is there association between safely managed sanitation and underfive children stunting? Therefore, we put the main interest (x) and output (y) indicator on this study title. Other factors are covariates variables.

Methodology:

• The authors should clearly describe how they selected the 16 townships from eight states and regions. They should also explain the sampling frame and sampling strategy used. The justification for selecting one ward in urban areas and two villages in rural areas should also be explained.

Ans: Township selection, sampling frame and ward/villages selection explanation has been revised in manuscript.

• The authors should cite a reference for the definition of stunting. The WHO's definition is a good option: "Stunting is defined as a child's height being more than two standard deviations below the median height for children of the same age and sex."

Ans: Thank. It has been updated and added the reference.

• The authors should cite a reference for the WHO/UNICEF Joint Monitoring Program (JMP) definition of safely managed sanitation.

Ans: Reference has been added.

• The authors should define improved sources of water or cite the WHO/UNICEF standard. The WHO/UNICEF standard is: "Improved water supply means that people have access to an improved drinking water source, such as a piped water supply, borehole, protected well, or rainwater collection."

Ans: Reference has been added.

Results:

• Table 2 should be shortened by focusing on biodata and sociodemographic factors.

Ans: Some factors (less focus) has been removed from table 2.

• The definitions of the variables in Table 2 should be clarified. For example, "adequate, inadequate, problematic promotion" should be defined.

Ans: Health literacy scores were classified in method section.

• The authors should explain why they did not include all of the variables in Table 2 in the multivariable analysis. It is possible that the association between safe sanitary situation and stunting would be different if all of the variables were included. For robust results, all variables should be included in the multivariable analysis.

Ans: According to the suggestion of statistician, the variables with crude odd ratio its pvalue more than 0.25 were not added on final maultivariate analysis model. I just revised to that paragraph to be more understanding to the reader.

• The authors should clarify whether the 95% confidence interval (CI) in Table 3 is for the crude OR or the adjusted AOR: the AOR = 0.53, while 95%CI = 1.11, 1.87

Ans. Thank you so much. CI value in Table is for AOR. 0.53 it is my typing error. I have already corrected to that value.

**Reviewer 2 comments:**

The title of the paper is appropriate and best suited. The literature review and problem statement justified the need for the study.

Ans: Thank for your positive comment and constructive feedback.

The methodology of stratified sampling needs justification; why sixteen townships, one urban ward, and two rural areas?

Ans: Two townships of 8 states were selected with randomly. Ward/village section has been revised on the method section.

Was the “preformed questionnaire” constructed by the authors or adopted? Please clarify.

Ans: It has been updated on Method section.

Statistical analysis and results drawn were sound and supported the existing area of sanitation and stunting in children.

Ans: Thank for your positive remark.

There are many grammatical and typographical errors in the text.

Ans: Thank a lot for your constructive feedback and valuable comment. Grammer has been checked and corrected.

Overall, we believe that these revisions address the reviewers' comments and significantly enhance the manuscript's quality. We have highlighted all the changes made in the revised manuscript. We sincerely hope that these changes demonstrate our commitment to improving the manuscript and that the revised version is now suitable for publication in PLOSONE. We look forward to your feedback and guidance on the next steps in the publication process.

Thank you for considering our revised manuscript.

Sincerely

Kittipong Sornlorm

Assistant Professor

Faculty of Public Health

Khon Kaen University, Thailand
